# Effective Poly (Cyclotriphosphazene-Co-4,4′-Sulfonyldiphenol)@rGO Sheets for Tetracycline Adsorption: Fabrication, Characterization, Adsorption Kinetics and Thermodynamics

**DOI:** 10.3390/nano11061540

**Published:** 2021-06-11

**Authors:** Muhammad Ahmad, Tehseen Nawaz, Mohammad Mujahid Alam, Yasir Abbas, Shafqat Ali, Muhammad Imran, Shuangkun Zhang, Zhanpeng Wu

**Affiliations:** 1 State Key Laboratory of Organic–Inorganic Composites, Beijing University of Chemical Technology, Beijing 100029, China; muhaahmad2-c@my.cityu.edu.hk (M.A.); ayasir@ymail.com (Y.A.); Zhangsk1988@sina.com (S.Z.); 2 Department of Mechanical Engineering, City University of Hong Kong, Kowloon, Hong Kong, China; 3 Department of Chemistry, The University of Hong Kong, Pokfulam, Hong Kong, China; tehsinshino@gmail.com; 4 Department of Chemistry, Faculty of Science, King Khalid University, Abha 61413, Saudi Arabia; malm@kku.edu.sa (M.M.A.); imranchemist@gmail.com (M.I.); 5 Guangdong Provincial Key Laboratory of Soil and Ground Water Pollution Control, School of Environmental Science and Technology, Southern University of Science and Technology, Shenzhen 518055, China; shafqat@sustech.edu.cn

**Keywords:** reduced graphene oxide, adsorption, tetracycline, thermodynamics

## Abstract

The development of excellent drug adsorbents and clarifying the interaction mechanisms between adsorbents and adsorbates are greatly desired for a clean environment. Herein, we report that a reduced graphene oxide modified sheeted polyphosphazene (rGO/poly (cyclotriphosphazene-co-4,4′-sulfonyldiphenol)) defined as PZS on rGO was used to remove the tetracycline (TC) drug from an aqueous solution. Compared to PZS microspheres, the adsorption capacity of sheeted PZS@rGO exhibited a high adsorption capacity of 496 mg/g. The adsorption equilibrium data well obeyed the Langmuir isotherm model, and the kinetics isotherm was fitted to the pseudo-second-order model. Thermodynamic analysis showed that the adsorption of TC was an exothermic, spontaneous process. Furthermore, we highlighted the importance of the surface modification of PZS by the introduction of rGO, which tremendously increased the surface area necessary for high adsorption. Along with high surface area, electrostatic attractions, H-bonding, π-π stacking and Lewis acid-base interactions were involved in the high adsorption capacity of PZS@rGO. Furthermore, we also proposed the mechanism of TC adsorption via PZS@rGO.

## 1. Introduction

Antibiotics have been used worldwide for the treatment of various diseases related to humans and animals [1]. Tetracycline (TC) is one of the most extensively used antibiotics, owing to its low toxicity with a broad spectrum of activity [2]. However, in recent years, increasing concern has been raised due to poor degradation through the metabolism. Consequently, residual TC is discharged to the environment through urine and feces, causing non-point pollution [2,3,4]. It also causes chronic toxicity, lower immunity, dysbacteriosis, liver damage and gastrointestinal reactions in human bodies; and also affects the aquatic photosynthetic species and indigenous microbial growth, which ultimately damages the food chain [5,6]. Therefore, it is the need of the hour to remove TC from the environment for a green future [7,8].

In this regard, various techniques such as electrochemical degradation, photochemical degradation, chemical oxidation, biodegradation and adsorption have been applied to remove TC [9,10,11,12,13]. Among these techniques, adsorption is a promising approach to eliminate the residual TC due to its efficiency and cost-effectiveness [14]. The adsorbents that have been successfully used for this purpose including polymeric materials [14], montmorillonite [7], smectite clay [15], alumina [16], diatomite [8] and carbon materials [17,18]. Polyphosphazenes are an important class of organic-inorganic hybrid polymers, comprising nitrogen and phosphorus atoms in a conjugated binding owing to their backbone stability, structural diversity, biocompatibility, biodegradability and ability to form hybrid molecules [19,20,21,22,23,24,25,26,27]. Poly (cyclotriphosphazene-co-4,4-sulfonyldiphenol) (PZS) is a copolymer of hybrid phosphazenes [28] containing an organic-inorganic structure, which makes it a promising candidate with an extensive range of potential applications, e.g., encapsulation [29], catalyst support [30], carbon material precursor [31], electrochemical features [32] and bio-medicinal applications [33]. Fu et al. [34] and Wei et al. [35] successfully adsorbed different toxic materials from aqueous media using PZS microspheres. The excellent adsorption capacity of these microspheres is related to their various binding modes such as H-bonding, electrostatic interactions, π-π stacking and group bindings. However, they exhibited less surface area while the adsorption process demands a high surface area of adsorbents. To overcome this issue, various carbon-based substrates such as multi-walled carbon nanotubes and graphene sheets were introduced to enhance the surface area [36].

Herein, for the first time, PZS@rGO (4–12%) was used to investigate the adsorption of TC. We further investigated the effect of various parameters such as pH, temperature, initial concentration of adsorbate and adsorbent dosage on its adsorption. The main objectives of the present study are as follows: (1) controlling the growth of the PZS sheet on rGO; (2) examine the adsorption performance of these materials for TC and find out the optimum adsorption measurements; (3) describe their kinetic and thermodynamic models; and (4) explore the possible adsorption mechanism of TC. The structure-property relationship was investigated, which included the H-bonding, electrostatic interactions, π-π stacking, and Lewis-acid base interactions between adsorbent and adsorbate.

## 2. Experimental Details

### 2.1. Materials

All reagents such as methanol, triethylamine (TEA), acetonitrile, ethanol, 4,4′-sulfonyldiphenol (BPS), octadecylamine (ODA), graphite oxide (GO) and petroleum ether were purchased from Beijing Chemical Co., Ltd (Beijing, China). Tetracycline was acquired from Macklin Biochemical Co., Ltd (Shanghai, China). Hexachlorocyclotriphosphazene (HCCP) was recrystallized from petroleum ether from sublimation. However, the remaining chemicals were used without further treatment.

### 2.2. Synthesis of PZS@rGO

**Figure 1:**
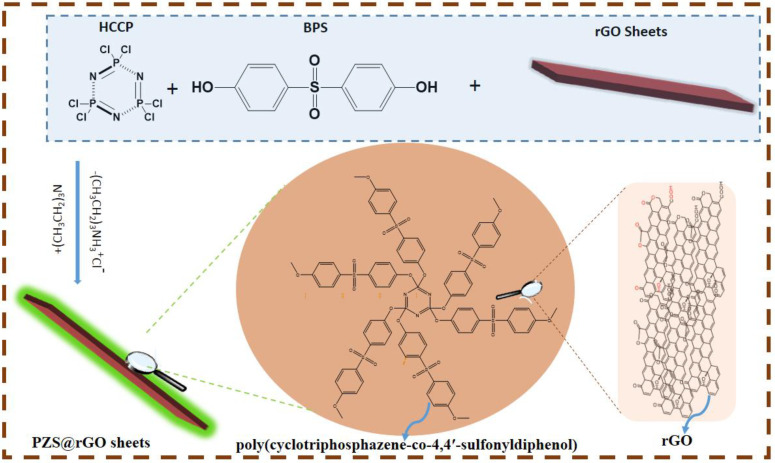
Scheme for the formation of PZS@rGO.

The scheme for the synthesis of the PZS@rGO is shown in Figure 1. We dispersed the prepared rGO (4%, 8% and 12%) [37] in 100 mL methanol under ultrasonication (150 W, 40 kHz) for 2 h. A total of 40 mg of HCCP and 80 mg of BPS solution in 10 mL methanol was mixed with the rGO solution. Afterward, 100 µL TEA was added dropwise, and magnetic stirring occurred for 7 h. The solution was washed with ethanol and then dried overnight under a vacuum. The PZS microspheres were prepared through analogous conditions reported elsewhere [19].

### 2.3. Adsorption of PZS@rGO Materials for Tetracycline Hydrochloride

Adsorption of Tetracycline hydrochloride was performed through the as-fabricated layered PZS@rGO by adding them into a conical flask containing 100 mL of TC solution (100 ppm). The conical flask was ultrasonically agitated for 40 s and placed in a thermostat at 30 °C with a rotation rate of 120 rpm (round per minute). In order to calculate the residual concentration of TC, we subsequently pipetted out 3 mL of suspension after a specific time interval, filtered it through a membrane (pore size: 0.22 μm) and finally, adsorption was measured at λ_max_ = 357 nm. Afterward, the adsorption capacity was calculated using Equation (1)

(1) $$q_{t}=\frac{(C_{0}-C_{t})V}{m}$$

where *q*_t_ is the adsorption capacity, *C*_0_ and *C*_t_ are the TC concentrations before and after adsorption (ppm), respectively, *V* is the volume of the solution (L), and *m* is the mass of adsorbent (g).

### 2.4. Characterization

UV-visible absorption spectra were obtained through a Lambda 3600 UV-vis spectrophotometer (PerkinElmer, Inc., Waltham, MA, USA). Further, X-ray photoelectron spectroscopy (XPS) analyses were performed via VG-ESCALAB 250 under a high vacuum (2 × 10^−9^ Pa), at a standard Al Kα source (1486.6 eV). The C 1s peak at 284.9 eV was taken as a reference for the binding energy. Fourier transform infrared (30 co-added) spectra were collected through a Bruker Vector-22 while implying KBr as a carrier to fix the samples. The surface morphology of the samples was examined via JEOL JSM-7610F field emission scanning electron microscope (SEM) (Tokyo, Japan). Transmission electron microscopy (TEM) images were obtained from the Hitachi-H-800 transmission electron microscope (TEM) (Tokyo, Japan). The surface area was calculated from nitrogen adsorption-desorption isotherms at 77 K through Micrometrics ASAP 2460 using the Brunauer−Emmett−Teller method. Prior to N_2_ adsorption-desorption, samples were subjected to 300 °C under vacuum for 6 h.

## 3. Results and Discussion

**Figure 2:**
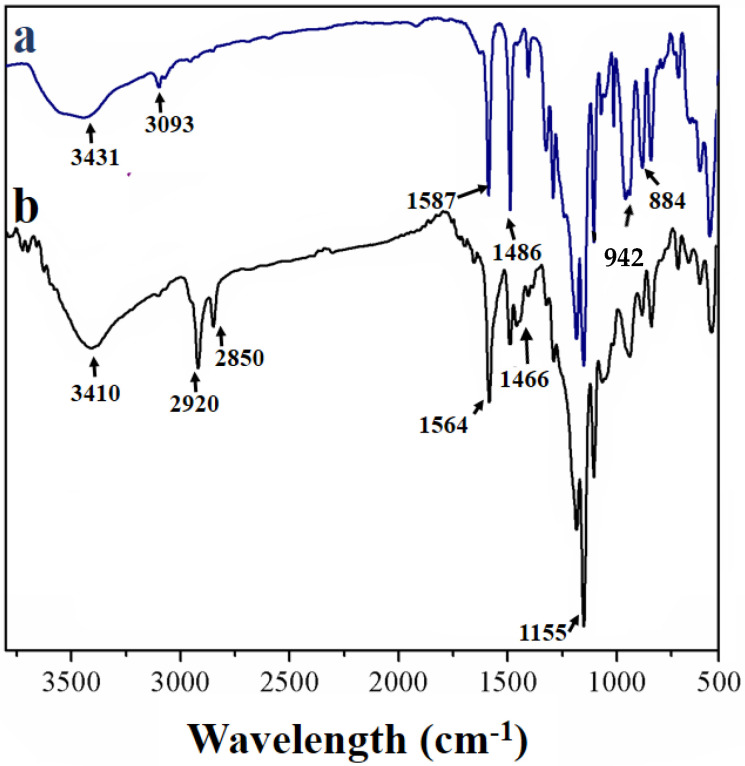
FTIR spectrum of (**a**) PZS and (**b**) PZS@rGO.

FTIR spectra of the PZS microsphere and PZS@rGO8% are shown in Figure 2. The characteristic peaks for hydroxylic (Phenolic group) could be observed at 3431 and 3093 cm^−1^ of the PZS microsphere (Figure 2a). The peaks at 1292 and 1152 cm^−1^ were attributed to O = S = O and P = N peaks, respectively. A new peak at 942 cm^−1^ can be attributed to P-O-(Ph), which was evidence to prove the successful condensation of HCCP and BPS. FTIR spectra of PZS@rGO8% (Figure 2b) followed the characteristic peaks of PZS microspheres and rGO. The new peaks emerged at 2920 and 2850 cm^−1^ due to the C-H stretching vibration, which belonged to rGO. The peaks at 1564 and 1466 cm^−1^ represented the N-H amide stretching and C-N bonding, respectively. The wide stretching of the peak at 3410 cm^−1^ is evidence of the presence of the abundant –OH group, which later proved to be the key site for adsorption.

**Figure 3:**
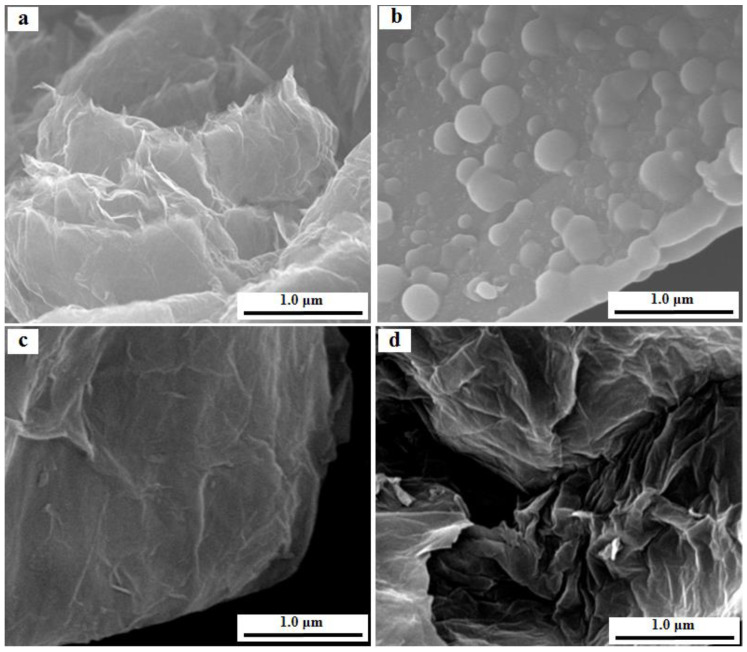
SEM images of (**a**) rGO, (**b**) PZS@rGO4%, (**c**) PZS@rGO8% and (**d**) PZS@rGO12%.

**Figure 4:**
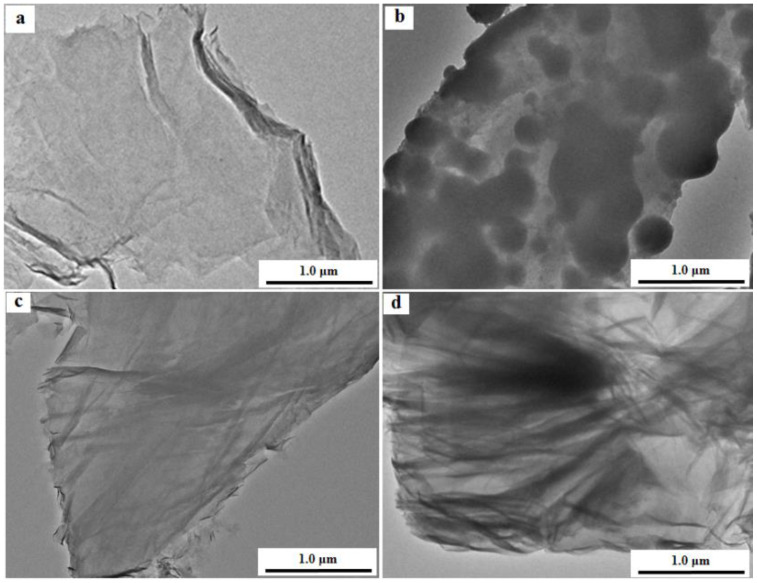
TEM images of (**a**) rGO, (**b**) PZS@rGO4%, (**c**) PZS@rGO8% and (**d**) PZS@rGO12%.

The morphology of PZS@rGO was examined through SEM (Figure 3) and TEM (Figure 4). SEM images of the PZS microspheres (Figure S1) show the spherical morphology, while the introduction of rGO in the PZS, shown in Figure 3a–d, exhibited the layered morphology. Moreover, PZS@rGO8% exhibited the fabrication of a smooth PZS layer over rGO sheets, while PZS@rGO4% showed the aggregation of PZS over rGO more likely in the form of microspheres, possibly due to less surface being offered by rGO due to its lower content. Meanwhile, the PZS@rGO12% morphology was more wrinkled due to the aggregation of rGO sheets. The TEM images of PZS@rGO (4%, 8% and 12%), Figure 4b–d, respectively, were consistent with the SEM results. The light grey area was attributed to the rGO sheets, while the dark area was indexed to PZS layers. PZS@rGO8% exhibited the thin layer of PZS equally spreading over rGO sheets. Meanwhile, PZS@rGO4% was aggregated in a bulk form, and PZS@rGO12% had excessive rGO that was aggregated.

**Figure 5:**
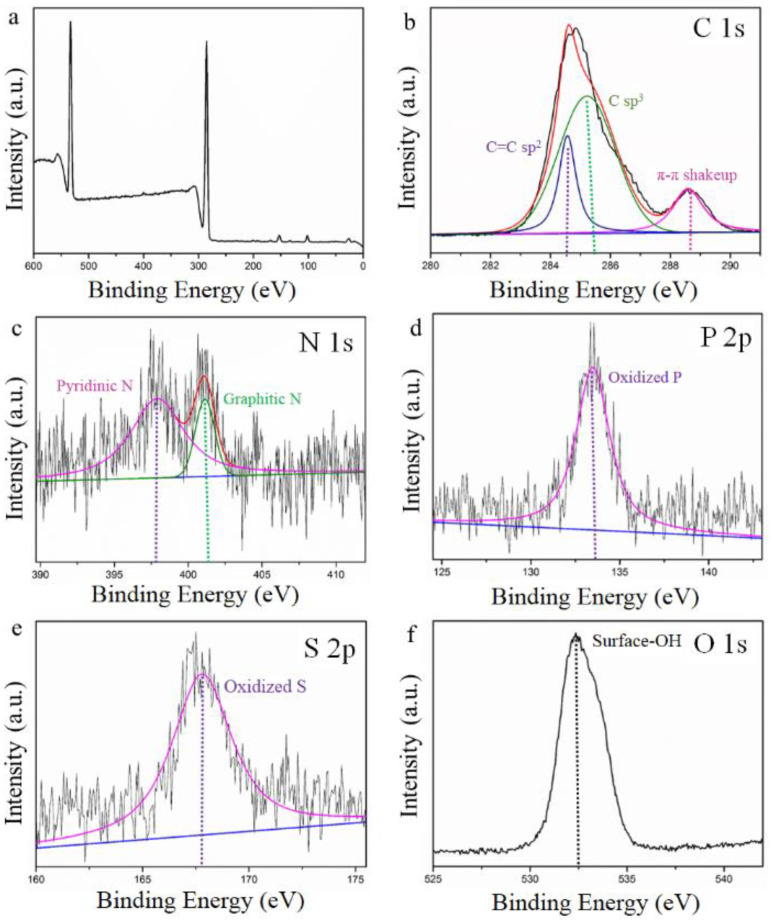
XPS spectra of (**a**) PZS@rGO8%, (**b**) C 1s, (**c**) N 1s, (**d**) P 2p, (**e**) S 2p and (**f**) O 1s.

Furthermore, the successful fabrication of PZS over rGO was evidenced by XPS (Figure 5). Figure 5a shows the complete XPS spectra of PZS@rGO8%, which revealed the all-necessary peaks of aforementioned adsorbent. The high-resolution C 1s XPS spectra of PZS@rGO (Figure 5b) was further deconvoluted into three components, C = C sp^2^ hybridized peak at 284.55 eV, C sp^3^ peak at 285.22 eV and the π-π stacking peak at 289.59 eV due to the conjugated system and aromatic structure [38]. The π-π stacking peak at 289.59 eV suggested the successful fabrication of PZS to rGO. Figure 5c shows the N 1s XPS spectra distributed into types of N species. Peaks at 397.94 and 401.1 eV were assigned to pyridinic N of PZS and graphitic N, respectively [39,40,41]. The XPS spectra of P 2p and S 2p were displayed in Figure 5d,e. Peaks at 133.52 and 167.5 eV were assigned to oxidized P 2p and S 2p, respectively [42,43]. The XPS spectra of O 1s (532.48 eV) shown in Figure 5f confirmed the presence of −OH species. Meanwhile, it was noticed from the XPS atomic content (Table S1) that PZS@rGO8% has 93.25% rGO content.

The surface area and structure of the PZS@rGO8% were explored by N_2_ absorption-desorption isotherms (Figure S2). PZS@rGO8% showed the type IV isotherms with H3-shaped hysteresis loops [44], which is a characteristic of mesoporous materials. The relative pressure of 0.451–1.0 is indexed to the mesoporous structure and the slit-shaped hole in the material [45]. The adsorbent exhibited a high surface area of 443 m^2^/g, which is considerably higher than the surface area of the PZS microsphere, 10.5 m^2^/g [35].

### 3.1. Adsorption Kinetics and Adsorption Capacity

**Figure 6:**
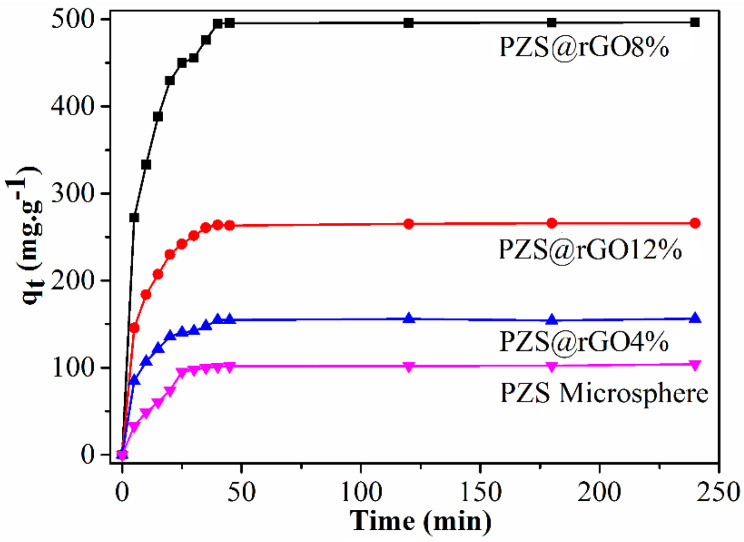
Adsorption capacity of different adsorbents at 100 ppm TC solution (temperature 30 °C).

Adsorption kinetics is a very important tool to probe adsorption behavior and elucidate the rate of adsorption. Kinetics data also describes significant information that might be helpful for the evaluation and understanding of adsorption mechanisms [46]. The adsorption capacity of the as-prepared PZS@rGO*x* (x = 4%, 8% 12%) and PZS microsphere versus time is disclosed in Figure 6. PZS@rGO8% exhibited the highest adsorption capacity of 496 mg/g, and equilibrium was attained in 40 minutes, which was higher than many reported studies Table S1. However, there were descending trends of 265 and 156 mg/g for PZS@rGO12% and PZS@rGO4%, respectively. Although the PZS microsphere contains all the elemental components required to perform adsorption at the same rate as its composites with rGO, it was the surface area that played a significant role in the adsorption capacity. The PZS@rGO8% adsorbent shows a higher surface area of 443 m^2^/g, which is a lot higher than the reported surface area of 10.5 m^2^/g of the PZS microsphere [35]. However, in the case of PZS@rGO4%, the PZS turned into an agglomerated morphology due to fewer rGO contents, and the rGO sheets tend to agglomerate in PZS@rGO12% (Figure 4).

The pseudo-first-order and pseudo-second-order kinetic models were used to explore the kinetic mechanisms of TC adsorption, and the linear forms of these models are shown in Equations (2) and (3), respectively.

(2) $$\log(q_{e}-q_{t})=\log q_{e}-\frac{k_{1}t}{2.303}$$

(3) $$\frac{t}{q_{t}}=\frac{1}{k_{2}q_{e}^{2}}+\frac{t}{q_{e}}$$

where *q*_e_ and *q*_t_ (mg g^−1^) are the adsorption capacity at equilibrium and at any time (*t*; min), respectively. *k*_1_ (min^−1^) is the pseudo-first-order rate constant and *k*_2_ (g mg^−1^ min^−1^) is the pseudo-second-order rate constant. The kinetic parameters and the correlation coefficients (R^2^) obtained by linear regression are listed in Table 1.

**Figure 7:**
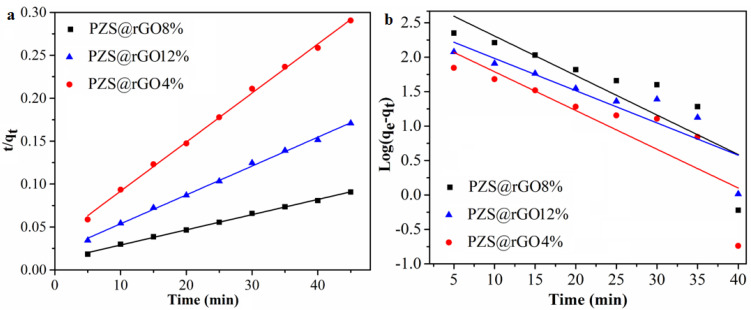
Linear fitting of Pseudo-second-order (**a**) and pseudo-first-order kinetics (**b**) for TC adsorption (C_0_ = 100 ppm).

According to Figure 7, a pseudo-second-order kinetic model better explains the adsorption of TC on PZS@rGO with a correlation coefficient approaching one (Table 1). Term *q*_e,exp_ is defined as the adsorption capacity at the equilibrium of any adsorbent from real-time experiments. While *q*_e,cal_ is known as the adsorption capacity at equilibrium calculated through different kinetic models, we may also refer to the latter term as theoretical adsorption. The theoretical adsorption capacity value differs with different applied kinetic models. The closer the theoretical adsorption capacity with real-time experimental results are, the more probable the adsorption system follows that particular kinetic model. In this case, theoretical adsorption capacity values suggest the adsorption system follows the pseudo-second-order reaction. Furthermore, the correlation R^2^ values of pseudo-second-order reaction are close to unity. The fact that the adsorption of TC through PZS@rGO followed the pseudo-second-order reaction confirms that there are different kinds of interactions between adsorbent and adsorbate due to the microporosity of PZS@rGO and surface functional groups of both adsorbent and adsorbate [47].

### 3.2. Adsorption Isotherms

The Langmuir and Freundlich isotherm models were applied to reveal insights into the adsorption phenomena. The Langmuir isotherm model assumes that the molecules are adsorbed in a monolayer configuration on the adsorbent surface where all the adsorption sites possess the same energy, so the adsorption of a species is identical [48,49,50].In comparison, the Freundlich isotherm model states multilayer, non-ideal adsorption, and the adsorption sites are unevenly distributed, involving a different affinity for adsorbing molecules. Equations (4) and (5) illustrated linear forms of Langmuir and Freundlich isotherm models, respectively.

(4) $$\frac{C_{e}}{q_{e}}=\frac{1}{bq_{m}}+\frac{C_{e}}{q_{m}}$$

(5) $$\log q_{e}=\log K_{f}+\frac{1}{n}\log C_{e}$$

where *b* is the adsorption energy constant (L mg^−1^), *q*_m_ is the theoretically calculated maximum Langmuir adsorption capacity (mg g^−1^), *C*_e_ is related to the equilibrium adsorption concentration (mg L^−1^) and *K*_f_ is associated to the Freundlich constant [(mg/g)·(L/mg) ^1/n^] while 1/*n* is the adsorption strength.

**Figure 8:**
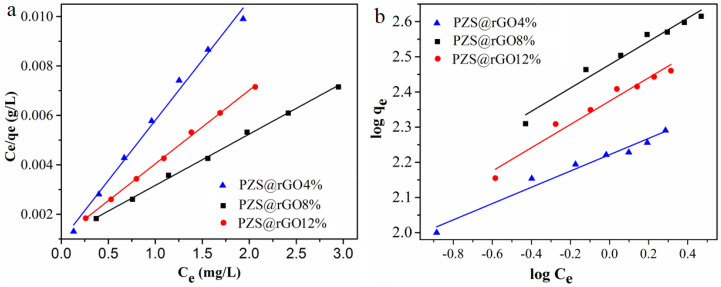
The adsorption isotherm using PZS@rGO*x* (x = 4%, 8% and 12%) adsorbents at 30 °C: Langmuir (**a**) and Freundlich model plots (**b**).

The adsorption of TC on PZS@rGO followed the Langmuir isotherm in comparison to Freundlich isotherm (Figure 8). At 303 K, the R^2^ value of adsorption of TC on PZS@rGO*x* was almost equal for the Langmuir and Freundlich equations, Table 2. This indicated that the adsorption of TC is more consistent with the Langmuir isotherm in comparison to the Freundlich isotherm and the adsorption of TC was monolayered.

### 3.3. Batch Experiments

For further experiments, PZS@rGO8% is used as the adsorbent as it has the maximum adsorption capacity, as shown in Figure 6.

#### 3.3.1. Effect of pH

**Figure 9:**
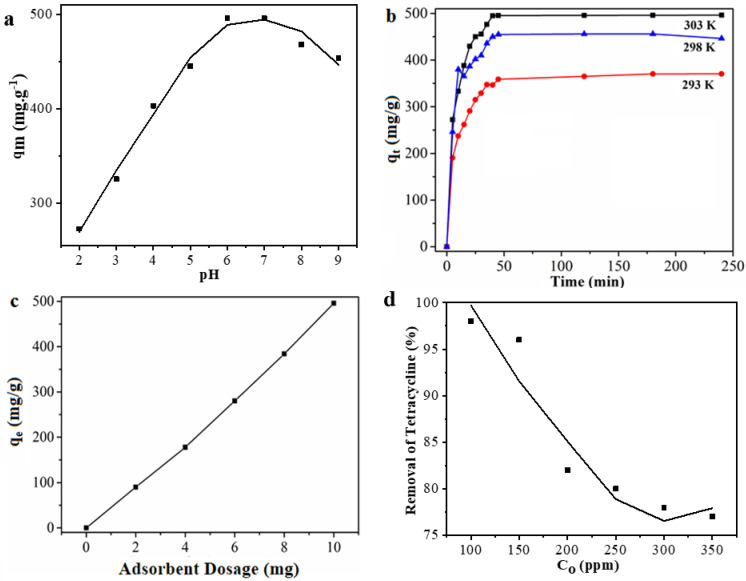
Effect of initial pH (10 mg PZS@rGO8% adsorbent, 100 ppm TC, volume of solution 100 mL, Temperature 30 °C) (**a**), effect of Temperature (10 mg PZS@rGO8% adsorbent, 100 ppm TC, volume of solution 100 mL, pH = 6) (**b**), effect of PZS@rGO8% adsorbent dosage (100 ppm TC, volume of solution 100 mL, pH = 6, temperature 30 °C) (**c**), effect of initial concentration (10 mg PZS@rGO8% adsorbent, volume of solution 100 mL, pH = 6, temperature 30 °C) (**d**).

The pH of a solution is an important factor that greatly influences the adsorption process because pH influences the surface charge and ionization behavior of materials. Figure 9a illustrates the effect of the initial pH of the solution on the adsorption capacity of PZS@rGO. The adsorption of TC was immensely lowered at pH = 2.0, and it could be related to the ion competition between H^+^ and TC^+^ for the adsorption sites of PZS@rGO [51]. The adsorption of TC increased with the increasing pH of the solution. The adsorption capacity reaches a maximum point at pH = 6.0. The ionization and hydration of TC may reduce, which is advantageous to the adsorption process via H-bonding and π–π stacking effect. TC may exist in a zwitter-ion formation at pH = 6–7. However, a further increase in pH slightly reduced the adsorption of TC. An increase in pH above a neutral value may affect H-bonding via OH^-^ generation [52]. Further experiments are conducted at the optimized pH = 6.

#### 3.3.2. Effect of Temperature

The temperature has a significant impact on the adsorption of TC, as shown in Figure 9b. We observe that at equilibrium, the adsorption capacity of PZS@rGO increases from 365 to 455 mg/g when the temperature is raised from 293 to 298 K. The results explain that the adsorption of TC is ideal at 303 K. As the temperature increases, the kinetic energy of molecules increases resulting in a higher proportion of molecular collision, ultimately increasing the amount of adsorption. According to Le Chateliar’s principle, when all the other conditions are constant, an increase in temperature shifts the equilibrium in the forward direction when the reaction is endothermic in nature, which was later confirmed by the thermodynamic data (Table 3). An increase in temperature enhances the kinetic energy of molecules, as the temperature is directly proportional to the kinetic energy, which ultimately enhances the mobility of molecules, and therefore, adsorption is increased [53]. Therefore, these results facilitate to opt for the best conditions to pursue the adsorption of TC via PZS@rGO for practical implications.

#### 3.3.3. Effect of Dosage

At the initial concentration, the effects of dosage on the amount of adsorption were studied, and the results are shown in Figure 9c. The removal of TC significantly increased with an increase in dosage amount. When the dosage of the adsorbent increased from 2 to 10 mg for 100 ppm initial concentration, the removal efficiency of the adsorbent increased from 90 to 496 mg/g, respectively. This is due to the adsorbent’s more active adsorption sites at high dosage concentrations.

#### 3.3.4. Effect of Initial Concentrations

The variation in adsorption capacity of PZS@rGO due to different initial concentrations of tetracycline was examined at pH = 6.0, 303 K and dosage of 0.1 g L^−1^. Adsorption of TC decreases with the increase in initial concentration of TC, as shown in Figure 9d. Typically, this phenomenon is related to the saturation of available adsorption sites, and with the increase in concentrate, PZS@rGO does not provide enough binding sites for TC adsorption. As the initial concentration increases from 100 to 300 ppm, the removal percentage of TC decreases from 98% to 77%, respectively, suggesting at lower initial concentrations of TC, the removal percentage is higher.

### 3.4. Adsorption Thermodynamics

The thermodynamic parameters of PZS@rGO are determined at 20, 25 and 30 °C, and adsorption isotherm constants were obtained. Gibbs free energy was used as a criterion to determine the spontaneity of reaction and was calculated by Equation (6).

ΔG = −RT ln KL(6)

where ΔG is the change in Gibbs free energy, R is the general gas constant (8.314 J/K mol), T is the temperature (K) and *K_L_* is the distribution coefficient of Langmuir equilibrium isotherm (L/mol). Change in Enthalpy (ΔH) and entropy (ΔS) were calculated using Equation (7):

ΔG = ΔH − TΔS(7)

All the thermodynamics data are shown in Table 3. Negative values of ΔG suggest the adsorption reaction is spontaneous while 30 °C is the most favorable temperature for adsorption reaction. A rise in temperature increases the diffusion rate and a reduction in the viscosity of the solution [54]. The low ΔS values exhibit no massive changes in the structure of the adsorption system, suggesting that there might be physical interactions more influential than chemical interactions [55]. Positive values of ΔH indicate that the adsorption process has to be endothermic in nature, and these results suggest the probability of bonding between adsorbent and dye [56]. If an endothermic reaction takes place, then for the process to remain spontaneous, the entropy change should not only be positive, but TΔS must exceed ΔH, numerically, so that the net Gibbs free energy change as a whole is negative. The trend of fewer enthalpy changes than entropy has been noticed in TC adsorption. Generally, the low enthalpy change is related to physisorption (<40 kJ/mol). An increase in temperature accelerates the amount of TC adsorption, which is consistent with the experimental results. The thermodynamics of TC adsorption suggests adsorption may occur at mild conditions due to lesser enthalpy change, low entropy and negative Gibbs free energy change.

### 3.5. Mechanism

**Figure 10:**
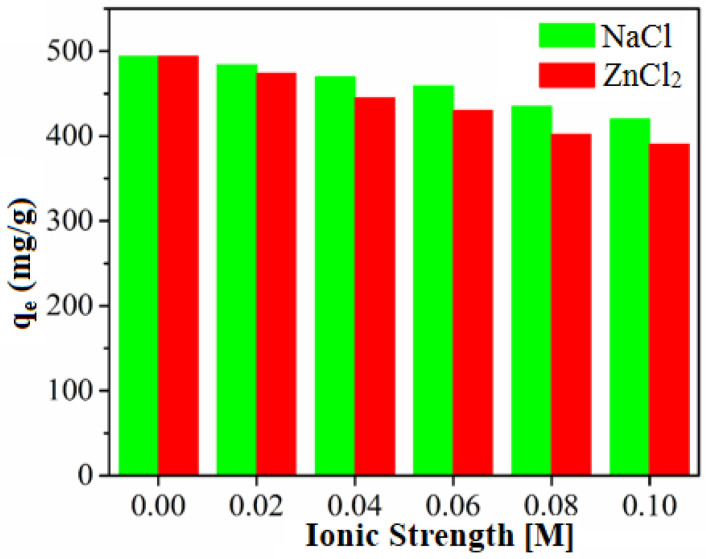
Effect of NaCl and ZnCl_2_ on adsorption system.

Based on these findings, we illustrated the adsorption mechanism of TC. The excellent adsorption capacity of PZS@rGO is dedicated to its high surface area and unique chemical composition. The BET surface area obtained by nitrogen adsorption-desorption (443 m^2^/g) was ca. 42 times higher than its microsphere [35]. The high surface area is the main difference between the PZS microsphere and PZS sheet’s adsorption capacity, as the chemical composition of both microspheres and sheets is similar. The results suggest that the introduction of rGO into PZS changed the morphology from microspheres into sheets and improved the adsorption capacity of the adsorbent. Considering the structural characterization of both PZS@rGO and TC, there were multiple factors involved during adsorption. The PZS sheets prepared in our study contain abundant hydroxyl groups on their surface, which was confirmed by FT-IR results (Figure 2). These hydroxyl groups have a tendency to produce an H-bonding interaction and an electrostatic interaction with TC. Further, it is a well-known electrostatic screening effect that the addition of salt inhibits the electrostatic interactions. The addition of NaCl in the adsorbent-adsorbate solution reduced the adsorption capacity shown in Figure 10. It is an obvious hint at the existence of electrostatic attraction. Both adsorbent and adsorbate possess a benzene ring in their structures, which enhances the π-π interaction [35].

**Figure 11:**
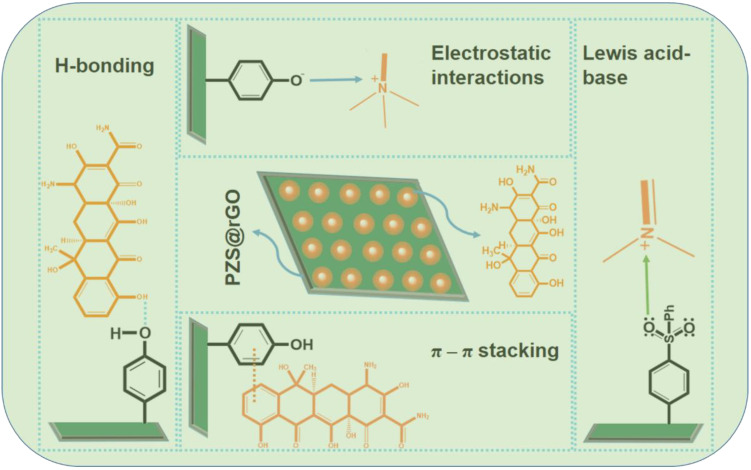
Proposed mechanism of TC decontamination through PZS@rGO.

Another route for adsorption mechanisms might be on the basis of acid-base interactions in Figure 11. TC contains a number of anionic, cationic and neutral groups in its structure, e.g., organic ammonium cations as the Lewis acid, while the PZS sheets have abundant nitrogen with a lone pair as the Lewis base. Later, it was confirmed by conducting an experiment by treating adsorbent with ZnCl_2_. Cyclotriphosphazenes have the ability to engage in chelation with Zn(II) [35]. Figure 10 showed the reduction in adsorption capacity of PZS@rGO after treating with ZnCl_2_ due to the occupation of the nitrogen lone pair by Zn (II) ions. Therefore, excellent adsorption of TC can be speculated to the high surface area of PZS@rGO, which facilitates adsorption by providing more sites for electrostatic interactions, π-π stacking and acid-base interactions.

## 4. Conclusions

In conclusion, the PZS layers modified with reduced graphene (PZS@rGO) have been successfully fabricated by simple co-precipitation methods under mild conditions. PZS@rGO successfully removed the tetracycline antibiotic from an aqueous solution and exhibited an excellent adsorption capacity of 496 mg/g in comparison to the PZS microsphere’s 102 mg/g. Further studies revealed that the adsorption of TC followed the Langmuir isotherm model and pseudo-second-order kinetics. Moreover, the removal of TC was spontaneous. The high adsorption capacity was attributed to a high surface area of the adsorbent. Along with a high surface area, electrostatic attractions, H-bonding, π-π stacking and Lewis acid-base interactions were involved for the high adsorption capacity of PZS@rGO. This study not only provides efficient drug removal but also highlights the effect of different parameters on the adsorption of TC over PZS@rGO.

## Figures and Tables

**Table 1:** Adsorption parameters for pseudo-first-order and pseudo-second-order models.

Adsorbents	C_0_ (ppm)	*q*_e,exp_	Pseudo-First-Order Model			Pseudo-Second-Order Model		
		(mg g^−1^)	*q*_e,cal_	*k*_1_	R^2^	*q*_e,cal_	*k*_2_	R^2^
			(mg g^−1^)	(min^−1^)		(mg g^−1^)	(g mg^−1^ min^−1^)	
PZS@rGO4%	100	155	223.87	0.12	0.685	175.13	9.4 × 10^−4^	0.998
PZS@rGO8%	100	496	761.18	0.13	0.711	565.97	2.77 × 10^−4^	0.998
PZS@rGO12%	100	265	282.87	0.107	0.769	297.61	5.161 × 10^−4^	0.998

**Table 2:** Adsorption parameters for Langmuir and Freundlich isotherms.

Adsorbents	Langmuir			Freundlich		
	*b* (L mg^−1^)	*q*_m_ (mg g^−1^)	R^2^	*K*_f_	*n*	R^2^
PZS@rGO8%	2.08	480	0.998	295	3.1	0.956
PZS@rGO12%	2.81	336	0.998	234	2.9	0.951
PZS@rGO4%	5.20	205.7	0.997	165.95	4.34	0.942

**Table 3:** Thermodynamics parameters for Adsorbents.

Samples	T/(K)	ΔG^0^/(kJ mol^−1^)	ΔS^0^/(kJ K^−1^ mol^−1^)	ΔH^0^s/(kJ mol^−1^)
PZS@rGO8%	293	−2.07	15.2	2.64
	298	−1.95		
	303	−1.84		

## Data Availability

Not applicable.

## Supplementary Materials

The following are available online at https://www.mdpi.com/article/10.3390/nano11061540/s1, Figure S1: SEM of PZS microspheres, Figure S2: N2 adsorption-desorption isotherm of PZS@rGO8%, Table S1: Comparison of the maximum equilibrium adsorption capacity of TC.
