# Supplementary material for: Effective Poly (Cyclotriphosphazene-Co-4,4′-Sulfonyldiphenol)@rGO Sheets for Tetracycline Adsorption: Fabrication, Characterization, Adsorption Kinetics and Thermodynamics
Source: Nanomaterials (Basel). 2021 Jun 11;11(6):1540. doi: 10.3390/nano11061540 (PMC8230582; doi:10.3390/nano11061540)
Supplement: Supplementary file 1 [file nanomaterials-11-01540-s001.zip › nanomaterials-1248160-supplementary.pdf]

Supporting Information

Effective Poly (Cyclotriphosphazene-Co-4,4′-Sulfonyldiphenol)@rGO Sheets for Tetracycline Adsorption: Fabrication, Characterization, Adsorption Kinetics and Thermodynamics

Muhammad Ahmad [1,2,†], Tehseen Nawaz [3,†], Mohammad Mujahid Alam [4], Yasir Abbas [1], Shafqat Ali [5],
Muhammad Imran [4], Shuangkun Zhang [1] and Zhanpeng Wu [1,*]

1 State Key Laboratory of Organic–Inorganic Composites, Beijing University of Chemical Technology, Beijing 100029, China; muhaahmad2-c@my.cityu.edu.hk (M.A.); ayasir@ymail.com (Y.A.); Zhangsk1988@sina.com (S.Z.)
2 Department of Mechanical Engineering, City University of Hong Kong, Kowloon, Hong Kong
3 Department of Chemistry, The University of Hong Kong, Pokfulam, Hong Kong; tehsinshino@gmail.com
4 Department of Chemistry, Faculty of Science, King Khalid University, Abha 61413, Saudi Arabia; malm@kku.edu.sa (M.M.A.); imranchemist@gmail.com (M.I.)
5 Guangdong Provincial Key Laboratory of Soil and Ground Water Pollution Control, School of Environmental Science and Technology, Southern University of Science and Technology, Shenzhen 518055, China; shafqat@sustech.edu.cn
* Correspondence: wuzp@mail.buct.edu.cn
† Muhammad Ahmad and Tehseen Nawaz contributed equally in this work.

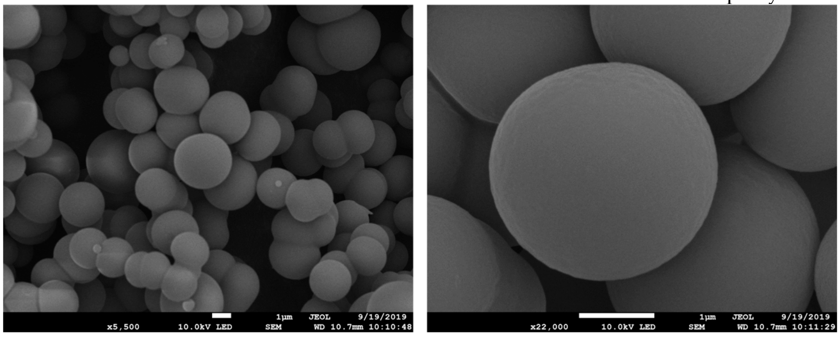

Figure S1. SEM of PZS microspheres

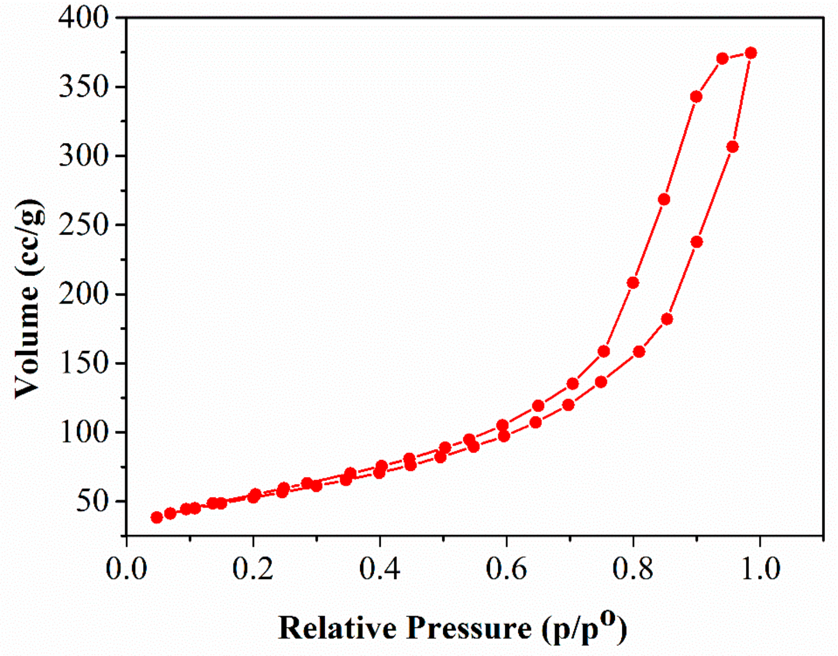

Figure S2. N_2 adsorption-desorption isotherm of PZS@rGO8%

Table S1. Comparison of the maximum equilibrium adsorption capacity of TC

Adsorbents	pH	T(K)	S_{BET} (m^2/g)	q_{max} (mg/g)	Refs
CuCo/MIL-101	4.8	318	2342	161.85	[1]
MWCNT/MIL-53	7	303	60.17	180.68	[2]
MOF/graphite oxide pellets	8	303	1266.69	228.00	[3]
Fe-based MOFs	-	-	1203	75.75	[4]
Polystyrene/graphene oxide	6	303	45.75	19.27	[5]

Zeolite Y	6.7	308	657.44	201.77	[6]
Cu-immobilized alginate	3	318	-	58.75	[7]
Alginate/graphene hydrogel	8	298	26.21	290.70	[8]
AG-ZIF	6	303	138.62	456.62	[9]
PZS@rGO8%	6	303	40	496	This work

References

[1] J. Jin, Z. Yang, W. Xiong, Y. Zhou, R. Xu, Y. Zhang, J. Cao, X. Li, C. Zhou, Cu and Co nanoparticles co-doped MIL-101 as a novel adsorbent for efficient removal of tetracycline from aqueous solutions, Science of the Total Environment, 650 (2019) 408-418.

[2] W. Xiong, G. Zeng, Z. Yang, Y. Zhou, C. Zhang, M. Cheng, Y. Liu, L. Hu, J. Wan, C. Zhou, Adsorption of tetracycline antibiotics from aqueous solutions on nanocomposite multi-walled carbon nanotube functionalized MIL-53 (Fe) as new adsorbent, Science of the Total Environment, 627 (2018) 235-244.

[3] L.-l. Yu, W. Cao, S.-c. Wu, C. Yang, J.-h. Cheng, Removal of tetracycline from aqueous solution by MOF/graphite oxide pellets: Preparation, characteristic, adsorption performance and mechanism, Ecotoxicology and environmental safety, 164 (2018) 289-296.

[4] D. Wang, F. Jia, H. Wang, F. Chen, Y. Fang, W. Dong, G. Zeng, X. Li, Q. Yang, X. Yuan, Simultaneously efficient adsorption and photocatalytic degradation of tetracycline by Fe-based MOFs, Journal of colloid and interface science, 519 (2018) 273-284.

[5] C.P. Okoli, A.E. Ofomaja, Development of sustainable magnetic polyurethane polymer nanocomposite for abatement of tetracycline antibiotics aqueous pollution: Response surface methodology and adsorption dynamics, Journal of cleaner production, 217 (2019) 42-55.

[6] M.M. Ali, M. Ahmed, B. Hameed, NaY zeolite from wheat (Triticum aestivum L.) straw ash used for the adsorption of tetracycline, Journal of cleaner production, 172 (2018) 602-608.

[7] X. Zhang, X. Lin, Y. He, Y. Chen, X. Luo, R. Shang, Study on adsorption of tetracycline by Cu-immobilized alginate adsorbent from water environment, International journal of biological macromolecules, 124 (2019) 418-428.

[8] Y. Zhuang, F. Yu, J. Ma, J. Chen, Enhanced adsorption removal of antibiotics from aqueous solutions by modified alginate/graphene double network porous hydrogel, Journal of colloid and interface science, 507 (2017) 250-259.

[9] Y. Kong, Y. Zhuang, K. Han, B. Shi, Enhanced tetracycline adsorption using alginate-graphene-ZIF67 aerogel, Colloids and Surfaces A: Physicochemical and Engineering Aspects, (2019) 124360.
